# The prognostic and predictive value of the lymphocyte to monocyte ratio in luminal-type breast cancer patients treated with CEF chemotherapy

**DOI:** 10.18632/oncotarget.8993

**Published:** 2016-04-26

**Authors:** Hongfei Ji, Qijia Xuan, Caichuan Yan, Tao Liu, Abiyasi Nanding, Qingyuan Zhang

**Affiliations:** ^1^ Department of Cancer Molecular and Biology, Cancer Institute, Harbin Medical University, Harbin, China; ^2^ Department of Medical Oncology, Tumor Hospital of Harbin Medical University, Harbin, China; ^3^ Department of Cancer Molecular and Biology, Heilongjiang Academy of Medical Sciences, Harbin, China; ^4^ Department of Pathology, Tumor Hospital of Harbin Medical University, Harbin, China

**Keywords:** breast cancer, lymphocyte-to-monocyte ratio, 5-fluorouracil, prognostic factor

## Abstract

Several reports have suggested that peripheral blood-based parameters are associated with host immunity response, which is an essential component of the pathogenesis and progression of cancer. The purpose of the present study was to identify the prognostic significance of various peripheral blood-based biomarkers and to determine the optimal cut-off value suitable for luminal breast cancer patients. We found that lymphocyte-to-monocyte ratio (LMR) was significant prognostic predictors. And the patients with a CEF regimen and LMR ratio ≥ 5.2 gained a good prognosis. This study suggested that the LMR could be regarded as an independent prognostic factor in luminal breast cancer patients. The elevated LMR level also had enhanced 5-fluorouracil sensitivity in luminal breast cancer patients.

## INTRODUCTION

Breast cancer is a complex and heterogeneous disease, and is categorized into three major subtypes based on gene expression profiles: luminal, basal like, and human epidermal growth factor 2 (HER2) enriched, which exhibit distinct clinical features, responses to specific chemotherapy, and prognosis [1–3]. Despite improvements in treatment, the morbidity and mortality rates in luminal breast cancer remain high [4–5]. Recent breakthroughs in cancer immunology substantiated that the host immune system correlates with cancer development and progression, and immunomodulating therapy has emerged as an effective novel therapeutic strategy [6–9]. Furthermore, the host immune system should be taken into account even during conventional chemotherapy treatment, as it has been found to influence the clinical response to chemotherapy.

Recent reports suggested that the peripheral blood-based parameters, such as absolute monocyte count (AMC), absolute lymphocyte count (ALC), neutrophil-to-lymphocyte ratio (NLR), lymphocyte-to-monocyte ratio (LMR), and platelet-lymphocyte ratio (PLR), are associated with host immunity response [10–14]. Moreover, there is a reliable correlation between the above parameters and increased survival time in a wide range of malignancies [15–19].

To the best of our knowledge, there are no comprehensive data available evaluating a set of peripheral blood-based biomarkers in luminal breast cancers. The purpose of the present study was to identify the prognostic significance of various peripheral blood-based biomarkers, and to determine the optimal cut-off value suitable for luminal breast cancer patients.

## RESULTS

### Patient and tumor characteristics

Data from two hundred and fifty-nine patients were collected for the analysis. The characteristics of the enrolled patients are generalized in Table 1. The patient median age was 48 years (range, 25–76 years). Over half of all patients (56.4%) were premenopausal and 29.3% had no lymphatic metastasis. The median tumor size was 4 cm. Twenty-five were histological grade I, 220 were histological grade II, and 14 were histological grade III. Two hundred and six (79.5%) were ER positive, and 222 (85.7%) were PR positive. Of the 259 cases, 216 (83.4%) were HER2 IHC level 0/1+, 38 (14.7%) were 2+, and 5 (1.9%) were 3+. 31.3% of breast tumors were luminal-A breast cancer. Of these, 128 breast cancers were ≥20% (49.4%) Ki-67 positive and 72 patients were P53 positive (27.8%).

**Table 1:** Characteristics of luminal breast cancer according to the lymphocyte-to-monocyte ratio

Characteristic	Overall(%)	LMR<5.2	LMR≥5.2	P-value
**Age**				
**≤50**	148 (57.1)	86	62	0.450^a^
**>50**	111 (42.9)	59	52	
**Menopause status**				
**No**	146 (56.4)	84	62	0.614^a^
**Yes**	113 (43.6)	61	52	
**Tumor size(cm)**				
**<2**	56 (21.6)	35	21	0.290^a^
**≥2**	203 (78.4)	110	93	
**Nodal status**				
**N0**	76 (29.3)	37	39	0.133^a^
**N+**	183 (70.7)	108	75	
**Histological grade**				
**I**	25 (9.7)	16	9	0.223^a^
**II**	220 (84.9)	124	96	
**III**	14 (5.4)	5	9	
**ER status**				
**ER+**	206 (79.5)	118	88	0.440^a^
**ER-**	53 (20.5)	27	26	
**PR status**				
**PR+**	222 (85.7)	120	102	0.153^a^
**PR-**	37 (14.3)	25	12	
**HER2 status by IHC**				
**0/1+**	216 (83.4)	126	90	0.175^a^
**2+**	38 (14.7)	16	22	
**3+**	5 (1.9)	3	2	
**Ki 67status**				
**<20%**	131 (50.6)	78	53	0.262^a^
**≥20%**	128 (49.4)	67	61	
**P53 status**				
**Positive**	72 (27.8)	42	30	0.677^a^
**Negative**	187 (72.2)	103	84	
**Luminal subtype**				
**Luminal A**	81 (31.3)	50	31	0.226^a^
**Luminal B**	178 (68.7)	95	83	
**Chemotherapy**				
**CEF**	82 (31.7)	41	41	0.229^a^
**TAC**	177 (68.3)	103	74	
**Lymphocyte count(10^9^/L)**	1.76 (0.4-5.3)*	1.59 (0.4-4)*	1.98 (0.9-5.3)*	0.000^b^
**Monocyte count (10^9^/L)**	0.41 (0.1-2.2)*	0.51(0.2-2.2)*	0.28 (0.1-0.5)*	0.000^b^

* Representing mean and range in the bracket; the mean LMR level was 5.4 (range, 0.3–27.7). LMR, lymphocyte-to-monocyte ratio; ER, estrogen receptor; PR, progesterone receptor; HER2, human epithelial receptor 2.

a Chi-square test by two-sided Pearson's exact test.

b Wilcoxon rank-sum test.

### Cutoff values for the LMR in luminal breast cancer patients

**Figure 1:**
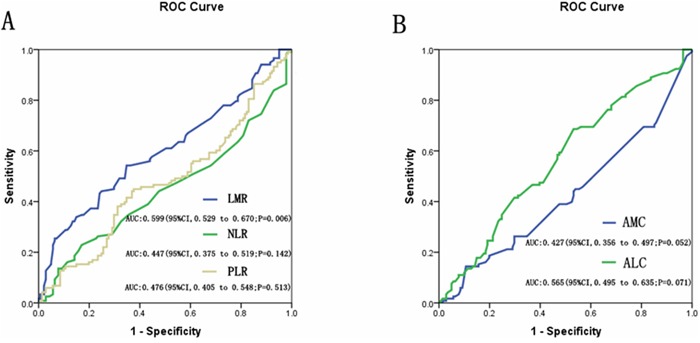
**A.** Receiver operating characteristics curves (ROC) and area under the curve (AUC) for LMR, NLR, and PLR in the study; **B.** Receiver operating characteristics curves (ROC) and area under the curve (AUC) for AMC and ALC in the study.

Receiver operating characteristics (ROC) curves and area under the curve (AUC) were used to determine the optimal cutoff points for the LMR, NLR, PLR, AMC, and ALC, based on their utility as markers for the clinical outcome of relapse, cancer-related death. Regarding the LMR in luminal breast cancer patients, 5.2 was identified as the optimal cutoff point for distinguishing good prognosis patients from poor prognosis patients (*P*=0.006) (Figure 1). There was no statistically significance in NLR, PLR, AMC, or ALC by ROC analyses (Figure 1).

The mean counts of lymphocytes were 1.76×10^9^/L (range, 0.4–5.3×10^9^/L) and the mean counts of monocytes were 0.41×10^9^/L (range, 0.1–2.2×10^9^/L) (Table 1). The mean LMR level was 5.4 (range, 0.3–27.7) (Table 1). All patients were also divided into either low- (<5.2) or high- (≥5.2) LMR groups. The mean lymphocyte count in the low-LMR and the high-LMR groups were 1.59×10^9^ and 1.98×10^9^, respectively (*P*<0.001) (Table 1). The mean monocyte counts in the low- and high-LMR groups were 0.51×10^9^ and 0.28×10^9^, respectively (*P*<0.001) (Table 1).

### Survival in terms of LMR status and chemotherapy

**Figure 2:**
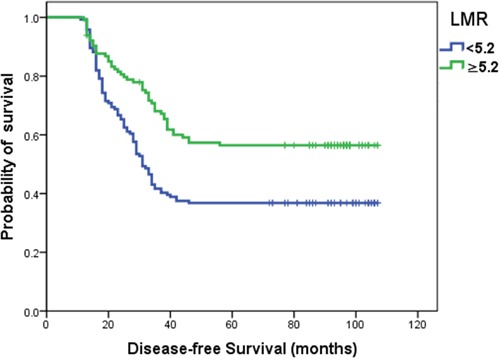
Kaplan–Meier curves for DFS according to optimal cutoff points of LMR

The optimal LMR cutoff point distinguished patients with a good prognosis. Kaplan–Meier survival curves analysis showed that the disease-free survival (DFS) in luminal breast cancer patients with a LMR ≥5.2 was significantly longer than those with a LMR <5.2 (*P*=0.001) (Figure 2).

**Figure 3:**
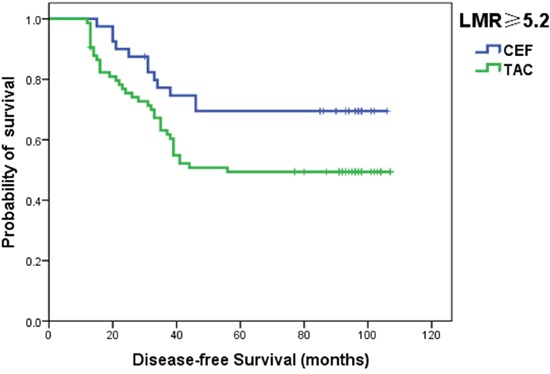
Kaplan–Meier curves for DFS in luminal patients with a LMR ≥5.2 according to the chemotherapy regimen

Patients with luminal breast cancer were also divided into two groups according to the chemotherapy regimens (CEF or TAC) after surgery. In luminal breast cancer patients with a LMR ≥5.2, Kaplan–Meier survival curves demonstrated that the DFS in patients with the CEF regimen was significantly longer than in those with the TAC regimen (*P*<0.05) (Figure 3).

### Univariate and multivariate analyses of clinicopathologic characteristics for DFS in luminal breast cancer

Results of univariate and multivariate analysis to identify prognostic factors for DFS in luminal breast cancer patients are shown in Table 2. When the correlation between clinicopathological variables and DFS were analyzed by univariate analysis, LMR [hazard ratio (HR) =0.560; 95% CI, 0.395–0.793; *P*=0.001], lymph node status (HR=1.748; 95% CI, 1.167–2.620; *P*=0.007), and chemotherapy (HR=1.676; 95% CI, 1.143–2.458; *P*=0.008) were significantly related to a higher risk of recurrence. Similarly, LMR and chemotherapy also showed a significant association with DFS by multivariate analysis.

**Table 2:** Univariate and multivariate analyses of clinicopathologic characteristics for DFS in luminal breast cancer

Variable	Univariate analysis		Multivariate analysis	
	HR(95% CI)	P-value	HR(95% CI)	P-value
LMR(<5.2; ≥5.2)	0.560(0.395-0.793)	**0.001**	0.582(0.408-0.831)	**0.003**
Lymph node status	1.748(1.167-2.620)	**0.007**	1.560(0.986-2.468)	0.057
Chemotherapy	1.676(1.143-2.458)	**0.008**	1.527(1.014-2.301)	**0.043**
Menopause status	0.827(0.594-1.153)	0.262	0.815(0.500-1.327)	0.410
Age	1.158(0.830-1.614)	0.389	1.118(0.683-1.830)	0.658
Tumor size	0.982(0.653-1.478)	0.931	0.852(0.556-1.305)	0.460
Histological grade status	0.996(0.635-1.560)	0.985	0.889(0.540-1.464)	0.645
Ki 67 status	1.055(0.757-1.469)	0.753	1.108(0.789-1.556)	0.552

Bold values are significant (P<0.05). DFS, disease-free survival; LMR, lymphocyte-to-monocyte ratio; HR, hazard ratio

### Expression of FAS in cell lines MCF-7 and T47D treated 5-fluorouracil and paclitaxel

**Figure 4:**
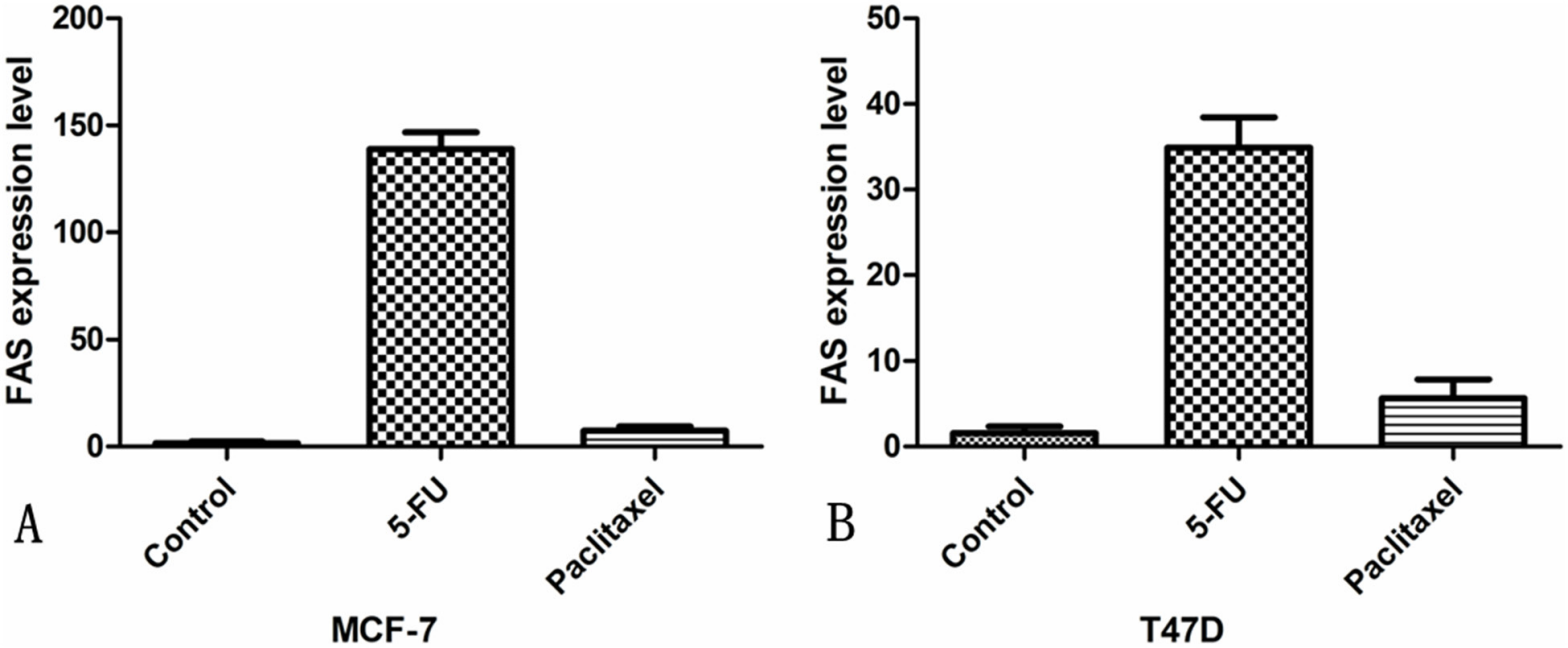
FAS expression level in breast cancer cell lines **A.** MCF-7 breast cancer cells; **B.** T47D breast cancer cells.

The MCF-7 and T47D cells are both luminal breast cancer cells. FAS expressions in 5-fluorouracil (5-FU) treated MCF-7 and T47D cell lines were higher than that in paclitaxel treated cell lines (*P*<0.01 and *P*< 0.01, respectively), as shown in Figure 4.

## DISCUSSION

In the present study, we performed a cohort study in 259 luminal breast cancer patients to evaluate the prognostic value of several peripheral blood-based biomarkers. We found that only the LMR was significantly associated with survival time in luminal breast cancer patients. Although there is limited evidence elucidating the mechanisms underlying the prognostic significance of the LMR, the lymphocytes and monocytes involved in host immune response may be a possible explanation [17, 23–26].

Lymphocytes and macrophages are the fundamental components of the tumor microenvironment [27–30]. For example, lymphocytes are pivotal mediators responsible for eliciting a positive immune response [28–32]. On the contrary, monocytes are known to infiltrate tumors and differentiate into tumor-associated macrophages, which are involved in tumor proliferation, invasion, metastasis, neovascularization, and recurrence [27-28, 33-34]. The advantage of the LMR index is that it is able to combine the information from lymphocytes and monocytes, and more comprehensively indicate the status of the host immune response.

In this study, we demonstrated that a LMR value of 5.2 was the optimal cutoff to predict DFS in luminal breast cancer patients. To the best of our knowledge, this is the first study to investigate an appropriate LMR cut-off value for luminal breast cancer. The tumor-infiltrating immune cells are highly heterogeneous between the different breast cancer subtypes [35–38]. As a result of this heterogeneity, the different breast cancer subtypes will induce different host immune responses, which will give rise to various LMR ratios depending on the breast cancer subtype. Consequently, this subtype-dependent LMR ratio may be responsible for the controversy surrounding the prognostic value of the LMR investigated by previous studies. Therefore, it is necessary to define a cut-off value for the LMR applied exclusively to the specific breast cancer subtypes, such as luminal breast cancer.

In addition, we found that the DFS was better in patients with a LMR ≥5.2 that received a CEF regimen, as compared with those receiving a TAC regimen. As we know, the distinguished difference between the CEF and the TAC regimen is 5-FU. In our study, we also found that 5-FU induced FAS expression in luminal breast cancer cells MCF-7 and T47D suggested that 5-FU could trigger tumor cell death through Fas/FasL dependent T-cell-mediated eradication. Hence, the improved effect of 5-FU was associated with higher levels of the LMR. Increasing evidence has demonstrated that 5-FU exhibits antitumor immunomodulatory activity by triggering host immune response and eliminating tumor cells, which is known as chemotherapy-induced immunogenic cell death (ICD). Specifically, 5-FU-induced ICD exerts its chemoimmunotherapeutic effects by recruiting innate or adaptive immune cells, enhancing cancer cell antigenicity, and releasing cytokine secretion [39–42]. The prerequisite to achieve ICD efficacy is a favorable immune system in patients, which means that the surrogate marker of host immune response, the LMR, needs to remain at an elevated level, and our results provided evidence to support this assumption.

In conclusion, our study identified an optimal cut-off for the LMR restricted to luminal subtype breast cancer. Using this LMR index, our results revealed that an elevated LMR could be regarded as an independent prognostic factor in luminal breast cancer patients. Moreover, our results indicated that the elevated LMR level enhanced the 5-FU sensitivity in luminal breast cancer patients. Therefore, the combination of lymphocyte-monocyte ratio and chemotherapy may be a promising strategy for anticancer treatments. As this was a retrospective and single-center study, further investigations need to be performed to validate our conclusion.

## MATERIALS AND METHODS

### Patients

This study, including the procedures for patient enrollment and recruitment, was approved by the Institutional Review Board of Tumor Hospital of Harbin Medical University and informed consent was obtained from each participant. The breast cancer patients were recruited between January 1st, 2006, and December 30th, 2008. The inclusion criteria were no metastatic lesions before surgery, infiltrating ductal carcinoma, ER/PR positive, and complete clinical and pathologic data. All patients in this study underwent an adjuvant chemotherapy regimen consisting of CEF (cyclophosphamide 600 mg/m^2^, day 1; epirubicin 60 mg/m^2^, day 1; and 5-FU 600 mg/m^2^, day 1; every 21 days for 6 cycles) or TAC (paclitaxel 175 mg/m^2^, day 1; doxorubicin 50 mg/m^2^, day 1; cyclophosphamide 500 mg/m^2^, day 1; every 21 days for six cycles). Depending on the lymph node and HER2 status of the breast cancer, patients were selectively given radiation and targeted therapy. Patients with positive ER or PR status received endocrine therapy (20 mg of tamoxifen per day) for 5 years. After surgery, every patient was followed up regularly until December 2014 or until death. Patients who developed disease relapse were confirmed by adequate diagnostic imaging modalities and pathology during the follow-ups.

### Methods

#### Blood sample analysis

The peripheral blood was collected in tubes containing ethylenediaminetetraacetic acid. The peripheral blood cells were counted by Sysmex XT-1800 Automated Hematology System (Shanghai, China). The peripheral LMR was calculated as the ratio of absolute counts between lymphocytes and monocytes. The peripheral NLR was translated as the ratio of absolute counts between neutrophils and lymphocytes. The peripheral PLR was interpreted as the ratio of absolute counts between platelets and lymphocytes. The peripheral blood samples were obtained after breast cancer diagnosis and before any treatment. All of the enrolled patients had no hematologic disorders or acute infections, implying that the cell counts could demonstrate the normal baseline value.

### Immunohistochemistry

All breast cancer tissues were formalin fixed and paraffin embedded. The immunohistochemical analysis was executed as described previously [20]. In brief, slides were incubated with an HER2 polyclonal antibody (Dako, CA, USA), P53 (1:500, DO-7; Dako), or Ki-67 (1:300, MIB-1; Dako). The stained tissues were evaluated according to the densities of staining and the number of stained cells. The samples were regarded as hormone receptor positive if cancer cells expressed an ER and/or PR greater than or equal to 10%. Tissues were considered positive for Ki-67 when more than 20% of the cells examined were stained. For P53, any staining of cells was deemed as P53 positive. The HER2 status was been evaluated according to the 2013 ASCO/CAP recommendations [21]. Each section was scanned at ×100 and ×400 magnification by microscope (Olympus BX51). Immunohistochemical (IHC)-based subtyping was determined according to the following definitions adopted by the 2011 St Gallen Consensus Panel: IHC-luminal (ER/PR+); IHC-luminal A (ER/PR+, HER2-, and Ki-67 index <14%); IHC-luminal B (ER/PR+, HER2-, and Ki67 index ≥14% or ER/PR+, HER2+) [3]. The other subtypes were not included in the study.

### Cell culture and treatment

MCF-7 and T47D cells were grown in in Roswell Park Memorial 1640 medium (RPMI 1640; Sigma-Aldrich; MO, USA) containing 10% fetal calf serum, 100 mg/ml streptomycin and 100 units/ml penicillin and incubated at 37°C supplemented with 5% of CO^2^. The cells were subcultured on 6-well plates for further experiments. After a 24h incubation to allow cell attachment, paclitaxel (0.2μM) or 5-FU (20μg/ml) were added to cells for 6h.

### Real-time PCR

Total RNA was extracted with the TRIzol reagent and cDNA was synthesized using a Prime-Script RT reagent kit (Takara Bio, Inc. Otsu, Shiga, Japan). The following PCR conditions were used: 1 cycle at 95°C for 10 min and then 40 cycles of 15 s at 95°C and 1 min at 60°C. Real-time PCR was performed on the ABI PRISM7500 Real-Time PCR System (Applied Biosystems) according to the manufacturer's instructions. The expression changes of the RNA was normalized to β-actin RNA. The sequences of PCR primers used were as follows: sense, 5′-GTGAGGGAAGCGGTTTACGA-3′and anti-sense, 5′- AGATGCCCAGCATGGTTGTT-3′ for Fas; sense, 5′-CCACGAAACTACCTTCAACTCC-3′ and anti-sense, 5′-GTGATCTCCTTCTGCATCCTGT-3′ for β-actin.

### Statistical methods

DFS was calculated as the time between diagnosis and disease relapse, or death from breast cancer. To evaluate whether increased LMR, NLR, PLR, AMC, or ALC correlated with prognosis of patients with breast cancer, we chose patients with disease relapse or cancer-related death during the follow-ups and defined them as the poor prognosis group. Other patients were included in the good prognosis group. ROC curves, and AUC, were performed to further evaluate the prediction status of LMR, NLR, PLR, AMC, and ALC. Sensitivity and specificity were calculated by the optimal cutoff points on the ROC curves, which decided the maximum value (sensitivity + specificity −1) of the Youden index [22].

The Chi-square or Fisher's exact tests were used for comparing clinicopathologic features between different LMR groups. Continuous variables, reported as lymphocyte and monocyte counts, were compared by the Wilcoxon rank-sum test. Risk factors of DFS were analyzed by univariate analysis with the log-rank test and multivariate analysis with the Cox proportional hazard model. The survival curves were performed by the Kaplan–Meier method and the comparisons between groups were assessed by the log-rank test. The results were showed as hazard ratios HRs and 95% CIs. The significance of the relative differential expression of FAS was determined by the two-tailed unpaired t test. Statistical significance was decided as *P*-values <0.05. All statistical analyses were performed using the IBM SPSS 20.0 software (IBM, USA).
